# Treatment of Infantile Paralysis

**Published:** 1892-01

**Authors:** 


### TREATMENT OF INFANTILE PARALYSIS.

Dr. Simon, the renowned Paris specialist in children's diseases, recommends the following treatment for infantile paralysis: At first counter-irritation along the vertebral column at the points corresponding to the roots of the paralyzed nerves. At the same time stimulate the functions of the skin by warm baths or vapor baths, given to the child in bed.

Chloral, aconite and conium are used to calm the nervous excitement.

After the first week electricity should form the basis of the treatment. Weak galvanic currents should be used, the negative pole being placed in a basin of water, into which the hand is plunged, while the positive pole is applied labile to the arm and shoulder. Length of treatment eight to ten minutes.

Later, faradism is to be used, but always with the greatest prudence.—W. F. R.—*Journal Nervous and Mental Diseases.*

